# Supplementary material for: Disseminating cells in human oral tumours possess an EMT cancer stem cell marker profile that is predictive of metastasis in image-based machine learning
Source: eLife. 2023 Nov 17;12:e90298. doi: 10.7554/eLife.90298 (PMC10781423; doi:10.7554/eLife.90298)
Supplement: Supplementary file 1. [file elife-90298-supp1.docx]

**Supplementary file 1 for Youssef et al.**

**Clinical details for first tumour cohort.**

| **Tumour** | **Nodal metastasis during 5 year follow up** | **Tumour site** | **Perineural spread** | **Depth of invasion** | **Tumour stage at presentation [TNM 7th Ed]** | **Tumour differentiation** | **Pattern of invasion** |
| --- | --- | --- | --- | --- | --- | --- | --- |
| 1 | yes | Left lateral tongue | yes | 11mm | pT3 pN2b | Moderate to poorly differentiated | dis-cohesive |
| 2 | yes | Right tongue / floor of mouth | No | 10mm | pT3 pN2b | Moderately differentiated | dis-cohesive |
| 3 | yes | Maxillary sulcus | No | 8.5mm | pT2 pNx | Moderately differentiated | dis-cohesive |
| 4 | yes | Right floor of mouth | No | 3mm | pT1 pNx | Moderate to poorly differentiated | dis-cohesive |
| 5 | yes | Left posterior ventro-lateral tongue and left tonsillar fossa | Yes | 7.2mm | pT4a pN2C | Moderate to poorly differentiated | dis-cohesive |
| 6 | yes | Left lateral border of tongue | Yes | 21mm | pT2 pN2b | Moderate to poorly differentiated | dis-cohesive |
| 7 | yes | Left tongue | Yes | 12mm | pT2 pN2b | Moderate to poorly differentiated | dis-cohesive |
| 8 | yes | Right buccal mucosa | Yes | 10mm | pT2 pN2b | Moderate to poorly differentiated | dis-cohesive |
| 9 | yes | Left tongue | Yes | 13.5mm | pT2 pN2b | Poorly differentiated | dis-cohesive |
| 10 | yes | Left buccal mucosa | yes | 11.5mm | pT2 pN2b | Moderate and focally poorly differentiated | cohesive |
| 11 | yes | Left tongue | yes | 11.5mm | pT2 pN2b | Moderate to poorly differentiated | dis-cohesive |
| 12 | No | Left tongue | yes | 7.9mm | pT2 pN0 | Poorly differentiated | dis-cohesive |
| 13 | No | Left buccal mucosa | No | 10mm | pT1 pNx, | Well to moderately differentiated | cohesive |
| 14 | No | Right tongue | No | 7.5mm | pT1 pN0 | Moderately differentiated | dis-cohesive |
| 15 | No | Left tongue | No | 5mm | pT1 pN0 | Moderately differentiated | dis-cohesive |
| 16 | No | Left tongue | No | 2.5mm | pT1 | Moderately differentiated | cohesive |
| 17 | No | Right soft palate | No | 6.5mm | pT1 | Moderate to poorly differentiated | dis-cohesive |
| 18 | No | Midline anterior mandibular gingiva | No | 4mm | pT4a pN0 | Well to moderately differentiated | dis-cohesive |
| 19 | No | Left mandibular alveolus | No | 13.5mm | pT4a pN0 | Moderately differentiated | dis-cohesive |
| 20 | No | Left dorsum of tongue | No | 1.5mm | pT1 | Well differentiated | cohesive |
| 21 | No | Right anterior tongue | No | 5mm | pT2 pNx | Moderate to poorly differentiated | cohesive |
| 22 | No | Left floor of mouth / ventral tongue | No | 3mm | pT1 | Well to moderately differentiated | cohesive |
| 23 | No | Right buccal mucosa | No | 3.5mm | pT1 | Moderately differentiated | cohesive |
| 24 | No | Lower right labial mucosa / vermilion | No | 3.5mm | pT1 | Moderately differentiated | dis-cohesive |
