# Supplementary material for: Disseminating cells in human oral tumours possess an EMT cancer stem cell marker profile that is predictive of metastasis in image-based machine learning
Source: eLife. 2023 Nov 17;12:e90298. doi: 10.7554/eLife.90298 (PMC10781423; doi:10.7554/eLife.90298)
Supplement: Supplementary file 2. [file elife-90298-supp2.docx]

**Supplementary file 2 for Youssef et al.**

**Clinical details and blinded analysis outcomes for second tumour cohort.**

| **Poor outcome cases** | | | | |
| --- | --- | --- | --- | --- |
| **Tumour** | **Tumour site** | **Clinicopathological details** | **TNM staging (TNM 7th edition default, 8th edition where indicated)** | **Correctly assigned in blinded analysis?** |
| 1 | Left retromolar trigone | Poorly differentiated with sarcomatoid pattern in part, widely dis-cohesive invasion pattern, multifocal, depth of invasion 14 mm, diameter 60 mm, focally present at mucosal margin, **metastatic squamous cell carcinoma** 5/62 right and left level II with ECS | pT3 pN3b (8th edition) / pT3 pN2c (7th edition) | Yes |
| 2 | Left posterior mandibular alveolus | Moderate to poorly differentiated, size 15 mm, depth invasion 5.5 mm, bony invasion with depth of invasion in bone 1.5 mm, cohesive, bony resection margin clear by 3 mm, mucosal margins clear by > 5 mm, **metastatic squamous cell carcinoma** in single lymph node left level Ib 33 mm no ECS, 1/48 | pT4a pN2a | Yes |
| 3 | Right lateral tongue | Moderate to poorly differentiated, size 28 mm, depth of invasion 7.7 mm, dis-cohesive with perineural spread, **metastatic squamous cell carcinoma** in four lymph nodes right levels IIa & IIb, largest deposit 42 mm and shows **ECS**, 4/35 | pT2 pN3b (8th edition) / pT2 pN2b (7th edition) | Yes |
| 4 | Right lateral tongue | Moderate to focally poorly differentiated, size 51 mm, depth of invasion 15 mm, cohesive invasion pattern, completely excised by > 5mm, **metastatic squamous cell carcinoma** in 2 lymph nodes right level II and junction of right levels II & III, largest deposit measures 18 mm no ECS, 2/54 | pT3 pN2b (8th edition) / pT4a pN2b (7th edition) | Yes |
| 5 | Left maxillary alveolus, hard palate and buccal mucosa | Moderately differentiated, size 49 mm, depth of invasion 28 mm. bony invasion of maxilla with involvement of maxillary antrum, multifocal perineural spread, LVI, cohesive invasion pattern, excised less than < 1 mm involved microscopically, **metastatic squamous cell carcinoma** in single left level I lymph node deposit measures 1.9 mm with no ECS | pT4a pN1(mi) (7th & 8th edition) | Yes |
| 6 | Left edentulous mandibular alveolus | Moderately differentiated, size 35 mm, depth of invasion 4.5 mm, bony invasion with depth of invasion in bone 2.5 mm, cohesive, excised by 3 mm, **metastatic squamous cell carcinoma** in single left level Ib lymph node, deposit measures 24 mm no ECS | pT4a pN1 (7th & 8th edition) | No |
| 7 | Right floor of mouth and ventrolateral tongue | Poorly differentiated, size 43 mm, depth of invasion 20.5 mm, multifocal perineural spread, intraneural spread and lymphovascular invasion, dis-cohesive invasion pattern, tumour present at anterior deep and lateral resection margins, **metastatic squamous cell carcinoma** present in 5 lymph nodes right levels I & I, largest deposit level I measuring 40 mm with **ECS**, lymphovascular emboli also seen in soft tissue of levels I & II, 5/8. Patient developed **local recurrence** in 2020 with invasion of mandible and overlying skin | pT3 pN3b (8th edition) / pT3 pN2b (7th edition) | Yes |
| 8 | Left dorsum of tongue | Moderately to poorly differentiated, size 27 mm, depth of invasion 9 mm, perineural spread, dis-cohesive invasion pattern, closely excised < 2 mm from several deep soft tissue resection margins, **metastatic squamous cell carcinoma** present in 2 lymph nodes left levels II & V, largest deposit measures 26 mm in left level II with **ECS** seen in both positive lymph nodes, 2/24 | pT2 pN3b (8th edition) / pT2 pN2b (7th edition) | Yes |
| 9 | Ventral surface of tongue, floor of mouth and lingual sulcus left > right | Moderate to poorly differentiated, size 45 mm, depth of invasion 24 mm, multifocal perineural spread, dis-cohesive invasion pattern, excised by 2 mm, **metastatic squamous cell carcinoma** present in 2 lymph nodes left levels Ib & II, the largest deposit measures 7 mm with **ECS** present in both lymph nodes, 2/71, **nodal recurrence** in neck left level Va 2020 treated with neck dissection - deposit measures 33 mm with ECS rypN3b | pT3 pN3b (8th edition) / pT4a pN2b (7th edition) | Yes |
| 10 | Right mandibular alveolus, right buccal sulcus and buccal mucosa | Moderate to poorly differentiated, size 81 mm, depth of invasion 31 mm, dis-cohesive invasion, bony invasion with destruction of lower border of mandible and direct extension into the soft tissue of right level I, into masseter muscle, positive margin in main resection specimen but further separate and intraoperative frozen sections clear, **metastatic squamous cell carcinoma** present in single right level right level Ib lymph node, the deposit measures 3 mm no ECS, 1/52. June 2020 **development of lung and bone metastases - July 2020 deceased** | pT4a pN1 (7th & 8th edition) | Yes |
| 11 | Anterior mandibular alveolus and anterior floor of mouth | Well to moderately differentiated, size = 45 mm, maximum depth of invasion 18 mm, bony invasion with depth of invasion in bone 9 mm, cohesive invasion pattern, excised very closely 0.9 mm, **metastatic squamous cell carcinoma** present in 2 lymph nodes left levels II - III & V, largest deposit spans left levels II - III measuring 32 mm with **ECS** seen in both deposits, 2/78 | pT4a pN3b (8th edition) / pT4a pN2b (7th edition) | No |
| 12 | Right mandibular alveolus, right buccal sulcus and buccal mucosa | Poorly differentiated with spindle cell / sarcomatoid areas, size 35 mm, depth of invasion 9.5 mm, invasion of bone and overlying skin with thickness of 50 mm, focally positive margin, **metastatic squamous cell carcinoma** present in 5 lymph nodes right levels Ib & II, largest deposit 26 mm with **ECS** seen in both levels, 5/29 | pT4a pN3b (8th edition) / pT4a pN2b (7th edition) | Yes |
| 13 | Right maxillary edentulous alveolar ridge | Moderate to poorly differentiated, size 19 mm, depth of invasion 4 mm with bony invasion, cohesive invasion pattern, excised by 3 mm. 3 months after surgery patient developed **nodal metastases** in right levels I - III, largest deposit measures 16 mm right level I, with **ECS** seen in right levels II, 4/45 | pT4a pNx nodal recurrence rpN3b (8th edition) / rpN2b (7th edition) | Yes |
| 14 | Right a anterior mandibular alveolus | Moderate to poorly differentiated, size 17 mm, depth of invasion 7 mm, dis-cohesive, LVI, PNS, tumour present at margins, bony invasion. 9 months later patient developed local nodal recurrence with **metastatic squamous cell carcinoma** present in 3 left level Ib lymph nodes the largest measures 27.5 mm and shows widespread **ECS**, 3/30 (19S00065398) | pT4a pNx, nodal recurrence rpN3b (8th edition) / rpN2c (7th edition) | Yes |
| 15 | Right posterior tongue | Moderate differentiated SCC, **metastatic squamous cell carcinoma** present in 4 lymph nodes right level I - III, largest deposit measures 80 mm with extensive **ECS**, 4/45 | pN3 (7th edition) / pN3b (8th edition) | Yes |
| 16 | Tongue base | Poorly differentiated, size 52 mm, depth of invasion at least 16 mm, LVS++, dis-cohesive invasion pattern, incompletely excised, metastatic squamous cell carcinoma present in 2 right level III lymph nodes largest deposit 6 mm no ECS, 2/27, local recurrence in 2020 | pT4a pN2b (7th & 8th edition) | Yes |
| 17 | Right posterior mandibular alveolus | Poorly differentiated, size 36 mm, depth of invasion 14 mm, LVS, dis-cohesive invasion pattern, excised by 3 mm, **metastatic squamous cell carcinoma** present in 4 lymph nodes, largest deposit 26 mm right level Ib no ECS, 4/41 | pT4a pN2b (7th & 8th edition) | Yes |
| 18 | Left buccal mucosa | Moderate to poorly differentiated, size 34 mm, depth of invasion 6.5 mm, dis-cohesive invasion pattern, completely excised by > 5 mm, metastatic squamous cell carcinoma in 1 left level I lymph node, deposit measures 20 mm no ECS, 1/36 | pT2 pN1 (7th & 8th editions) | No |
| 19 | Left retromolar trigone | Moderate to poorly differentiated, size 36 mm, depth of invasion 23 mm, bony invasion posterior mandible, LVS, PNS, dis-cohesive invasion pattern, closely excised by 1.7 mm, **metastatic squamous cell carcinoma** present in 4 lymph nodes left levels Ib & II largest deposit 26 mm with **ECS**, 4/40 | pT4a pN3b (8th edition) / pT4a pN2b (7th edition) | Yes |
| 20 | Right posterior mandibular alveolus | Poorly differentiated, size 64 mm, depth of invasion 31 mm, bony invasion, PNS, LVS, dis-cohesive invasion pattern, closely excised by 1.9 mm, **metastatic squamous cell carcinoma** present in 3 right level Ib lymph nodes, largest deposit measures 9 mm with focal minimal **ECS**, 3/31 | pT4a pN2b (7th & 8th edition) | Yes |
| 21 | Right mandibular alveolus | Moderate to poorly differentiated, size 44 mm, depth of invasion 27 mm, PNS, dis-cohesive invasion pattern, bony invasion, metastatic squamous cell carcinoma present in single lymph node 6 mm no ECS, 1/18 | pT4a pN1 (7th & 8th edition) | Yes (in both duplicates) |
| 22 | Right retromolar trigone | Moderate to poorly differentiated, size 22 mm, depth of invasion 7.5 mm, LVS, dis-cohesive invasion pattern, no bony invasion, completely excised by 5 mm, **metastatic squamous cell carcinoma** present in 3 lymph nodes right levels Ib & IIa, 26 mm right level Ib, no ECS, 3/49 | pT2 pN2b (7th & 8th editions) | Yes |
| 23 | Right retromolar trigone | Moderate to poorly differentiated, size 52.5 mm, depth of invasion 16 mm, PNS, dis-cohesive invasion pattern, bony invasion, closely excised by 1 mm, **metastatic squamous cell carcinoma** present in 4 left level Ib lymph nodes, largest deposit measures 12 mm with **ECS**, 4/23 | pT4a pN2b (7th & 8th edition) | Yes |
| 24 | Right lateral tongue | Well to moderately differentiated, size = 30.5 mm, depth of invasion 10.5 mm, dis-cohesive invasion pattern, microscopically involved margin, no metastatic squamous cell carcinoma present in neck dissection but in 2012 patient developed **loco-regional recurrence** in right neck submental / mandibular region and in 2013 contralateral level IV lymph nodes confirmed by FNA as SCC | pT2 pN0 - original resection | Yes |
| 25 | Hard palate | Moderate to poorly differentiated, size 45 mm, depth of invasion 12 mm, dis-cohesive invasion pattern, PNS, bony invasion, completely excised, 0/12, **Significant locoregional disease progression with invasion of pterygoid fossa, inferior orbital fissure and pterygopalatine fossa** | pT4a pN0 | Yes (in both blocks) |
| 26 | Left tongue | Moderate to poor, 26 mm, depth of invasion 8 mm, PNS, widely dis-cohesive invasion pattern, excised by 4 mm, **metastatic squamous cell carcinoma** present in 1 lymph node level III, 4.5 mm no ECS, 1/21 | pT2 pN1 (7th & 8th editions) | Yes |
| 27 | Right floor of mouth | Moderate to poor, size 29 mm, depth of invasion 12.5 mm, PNS, dis-cohesive invasion pattern, excised by 4 mm, **metastatic squamous cell carcinoma** present in a single lymph node level II measures 4 mm and shows minimal **ECS**, 1/15 | pT2 pN1 (7th & 8th editions) | Yes |
| **Good outcome cases** | | | | |
| **Tumour** | **Tumour site** | **Clinicopathological details** | **TNM staging (TNM 7th edition default, 8th edition where indicated)** | **Correctly assigned in blinded analysis?** |
| 28 | Right lateral tongue | Moderate to poorly differentiated, size 36 mm, maximum depth of invasion 2 mm, dis-cohesive invasion pattern, excised by 2 mm from inferior mucosal margin, 0/49 | pT2 pN0 | Yes (in both blocks) |
| 29 | Anterior mandibular alveolus | Well differentiated, size 27 mm. depth of invasion 4 mm, cohesive invasion pattern, excised by 2.5 mm | pT2 pNx | Yes (in both blocks and in both duplicates) |
| 30 | Right floor of mouth | Moderately differentiated, size 5 mm, depth of invasion 6 mm, foci suspicious of perineural spread, excised by 3.5 mm | pT1 pNx | Yes |
| 31 | Left lateral tongue | Moderately differentiated, size 21 mm, depth of invasion 6 mm, dis-cohesive invasion pattern, perineural spread, no lymphovascular invasion, excised by 3.5 mm, 0/26 | pT2 pN0 | Yes |
| 32 | Left buccal mucosa | Moderate to poorly differentiated, size 28 mm, depth of invasion 14.5 mm, discohesive invasion pattern, excised by 3 mm, 0/25 | pT2 pN0 | Yes (in both blocks and both duplicates) |
| 33 | Anterior mandibular alveolus | Moderately differentiated, size 22 mm, depth of invasion 10.5 mm, cohesive invasion pattern, invasion of body of mandible, excised by 2.5 mm, 0/36 (bilateral neck dissection) | pT4a pN0 | Yes (in both duplicates) |
| 34 | Left anterior lateral tongue | Well to moderately differentiated, size 30 mm, depth of invasion 12 mm, cohesive invasion pattern, excised by 2 mm from deep margin, 0/17 | pT2 pN0 | Yes |
| 35 | Left floor of mouth | Moderate to poorly differentiated, size = 25 mm, depth of invasion 2.5 mm, excised by 1 mm from a buccal gingival resection margin, 0/37 | pT2 pN0 | Yes |
| 36 | Right mandibular alveolus and right floor of mouth | Moderately differentiated, size 35 mm, depth of invasion 6.5 mm, discohesive invasion pattern, bony invasion, completely excised by > 5 mm | pT4a pNx | Yes |
| 37 | Anterior maxillary alveolus | Well to moderately differentiated, size 23 mm, depth of invasion 14 mm, cohesive invasion pattern, bony invasion, completely excised by > 5 mm | pT4a pNx | Yes |
| 38 | Left lower buccal sulcus | Well differentiated, size 42 mm, depth of invasion 3 mm, cohesive invasion pattern, excised by > 5 mm, no bony invasion, 0/11 | pT2 pN0 | Yes |
| 39 | Anterior maxillary alveolus | Moderately differentiated, size 23 mm, depth of invasion not recorded, bony invasion present, cohesive invasion pattern, completely excised by > 5 mm | pT4a pNx | No |
| 40 | Right mandibular alveolus and right floor of mouth | Moderately differentiated, size 27 mm, depth of invasion 8 mm, cohesive invasion pattern, excised by 2 mm, no invasion of mandibular rim resection | pT2 pNx | Yes |
| 41 | Left buccal mucosa | Moderately differentiated, size 17 mm, depth of invasion 6 mm, discohesive invasion pattern, excised by 4 mm from deep margin, 0/20 | pT1 pN0 | Yes |
| 42 | Left mandibular alveolus and buccal mucosa | Moderately differentiated, size 41.5 mm, depth of invasion 34 mm, cohesive invasion pattern, bony invasion, excised by 2 mm, 0/13 | pT4a pN0 | Yes |
| 43 | Left ventral surface of tongue | Moderately differentiated, size 21 mm, depth of invasion 6 mm, cohesive invasion pattern, excised by 1 mm from medial and deep soft tissue resection margins, 0/9 | pT2 pN0 | Yes (in both duplicates) |
| 44 | Right floor of mouth | Moderate to poorly differentiated, size 25.5 mm, depth of invasion 8.5 mm, widely discohesive, excised by 3 mm, no bony invasion of mandible, 0/21 | pT2 pN0 | Yes |
| 45 | Right lateral tongue | Moderate to poorly differentiated, size 19 mm, depth of invasion 8 mm, discohesive, widespread perineural spread and intraneural spread, widely dis-cohesive, excised by 2 mm from deep resection margin, 0/20 | pT1 pN0 | Yes |
| 46 | Hard palate | Moderate to poorly differentiated, size 47 mm, depth of invasion 19 mm, focal perineural spread, cohesive invasion pattern, invasion through maxilla into antrum, excised by 3 mm, 0/31 | pT4a pN0 | Yes |
| 47 | Right lateral tongue | Moderate to poorly differentiated, size 24 mm, depth of invasion 7 mm, perineural and intraneural invasion, dis-cohesive invasion pattern, completely excised by > 5 mm, 0/21 | pT2 pN0 | Yes |
| 48 | Right mandibular alveolus | Moderate to poorly differentiated, size 45 mm, depth of invasion 24.5 mm, perineural invasion, bony invasion of body of mandible, excised by 2 mm, 0/23 | pT4a pN0 | Yes |
| 49 | Right mandibular alveolus | Moderately differentiated, size 46 mm, depth not documented, dis-cohesive invasion pattern, bony invasion, excised by 1.5 mm, 0/38 | pT4a pN0 | Yes |
| 50 | Left lateral tongue | Moderately differentiated, size 26 mm, depth of invasion 10.5 mm, dis-cohesive invasion pattern, excised by 2.5 mm, 0/22 | pT2 pN0 | No |
